# Unique Versus Redundant Functions of IL-1α and IL-1β in the Tumor Microenvironment

**DOI:** 10.3389/fimmu.2013.00177

**Published:** 2013-07-08

**Authors:** Elena Voronov, Shahar Dotan, Yakov Krelin, Xiaoping Song, Moshe Elkabets, Yaron Carmi, Peleg Rider, Idan Cohen, Marianna Romzova, Irena Kaplanov, Ron N. Apte

**Affiliations:** ^1^The Shraga Segal Department of Microbiology, Immunology and Genetics, The Faculty of Health Sciences, Ben Gurion University of the Negev, Beer Sheva, Israel

**Keywords:** IL-1, carcinogenesis, tumor invasiveness, tumor-host interactions, immunogenicity, anti-tumor immunity, immunotherapy

## Abstract

Interleukin-1 (IL-1) is a major “alarm” upstream pro-inflammatory cytokine that also affects immunity and hematopoiesis by inducing cytokine cascades. In the tumor arena, IL-1 is produced by malignant or microenvironmental cells. As a pleiotropic cytokine, IL-1 is involved in tumorigenesis and tumor invasiveness but also in the control of anti-tumor immunity. IL-1α and IL-1β are the major agonists of IL-1, while IL-1Ra is a physiological inhibitor of pre-formed IL-1. In their secreted form, IL-1α and IL-1β bind to the same receptors and induce the same biological functions, but IL-1α and IL-1β differ in their compartmentalization within the producing cell or the microenvironment. IL-1β is only active in its processed, secreted form, and mediates inflammation, which promotes carcinogenesis, tumor invasiveness, and immunosuppression, whereas IL-1α is mainly cell-associated and in the tumor context, when expressed on the cell membrane, it stimulates anti-tumor cell immunity manifested by tumor regression. In the tumor milieu, extracellular levels of IL-1α are usually low and do not stimulate broad inflammation that promotes progression. Immunosuppression induced by IL-1β in the tumor microenvironment, mainly through MDSC induction, usually inhibits or masks anti-tumor cell immunity induced by cell-associated IL-1α. However, in different tumor systems, redundant or unique patterns of IL-1α and IL-1β expression and function have been observed. Recent breakthroughs in inflammasome biology and IL-1β processing/secretion have spurred the development of novel anti-IL-1 agents, which are being used in clinical trials in patients with diverse inflammatory diseases. Better understanding of the integrative role of IL-1α and IL-1β in distinct malignancies will facilitate the application of novel IL-1 modulation approaches at the bedside, in cancer patients with minimal residual disease (MRD), as an adjunct to conventional approaches to reduce the tumor burden.

## Expression and Secretion of IL-1α and IL-1β – The Major IL-1 Agonistic Molecules

The IL-1 family consists of 11 agonist and antagonist molecules that are centrally involved in regulating inflammatory responses. These include IL-1α, IL-1β, IL-1Ra, IL-18, IL-33, IL-1Ra, IL-36α, IL-36β IL-36γ, and IL-38 [reviewed in Ref. (1–7)]. Here, we will mainly focus on the two major IL-1 agonistic molecules, i.e., IL-1α and IL-1β, and IL-1 receptor antagonist (IL-Ra), which is a physiological inhibitor of IL-1 signaling.

IL-1α and IL-1β are synthesized as precursors of 31 kDa that are further processed by proteases to their mature secreted 17 kDa forms. IL-1 differs from most other cytokines by lack of a signal sequence, thus not passing through the endoplasmic reticulum-Golgi pathway; its mechanisms of secretion are not yet completely understood. IL-1Ra, which has a signal peptide, is secreted in the ER-Golgi exocytic pathway. Generally, IL-1 is produced and secreted by various cell types upon inflammatory or stress conditions, predominantly by myeloid cells, which display the strongest capacity to produce and secrete IL-1. Stimulation of IL-1 production occurs through signaling of Toll-like receptors (TLRs), which recognize conserved microbial molecules of pathogens, i.e., pathogen-associated molecular patterns (PAMPs) [reviewed in Ref. (8–10)] as well as endogenous molecules, which are products of damaged cells, termed danger-associated molecular patterns (DAMPs) [reviewed in Ref. (11–13)]. The activation of TLR signaling via the NF-κB pathway leads to the generation of many cytokines; IL-1 is a central cytokine produced by this pathway. Signaling through surface IL-1Rs and most of the TLRs is common and converges from MyD88 to NF-κB activation and induction of an inflammatory response, including expression of IL-1.

### Processing of IL-1β

The IL-1β-converting enzyme (ICE), or caspase-1, is a cysteine protease, that is activated in the cytosol on the inflammasome platform, and subsequently cleaves the inactive precursors of IL-1β, IL-18, and IL-33 into their mature secreted forms (2, 3, 14–18).

### Processing of IL-1α

The precursor of IL-1α (ProIL-1α) is processed by the Ca^2+^-dependent protease calpain into the mature 17 kDa form and the 16 kDa N-terminal cleavage product – the propiece of IL-1α, also termed IL-1α N-terminal peptide (IL-1NTP). The latent form of calpain is activated in cells under inflammatory conditions and especially upon loss of plasma membrane integrity, which occurs during necrosis (19). However, intracellular ProIL-1α is present in many cells because they contain calpain inhibitors and are thus unable to process and secrete IL-1α. Other proteases, such as elastase, chymase, or Granzyme B can also process ProIL-1α into smaller molecules with high inflammatory potential (20–22). Recently some involvement of the inflammasome in IL-1α secretion has been demonstrated (23–25). A biologically active membrane-associated form of IL-1α (23 kDa), which is anchored to the membrane via a mannose-like receptor, has been demonstrated in activated cells that express the cytokine. However, it is not clear how IL-1α is inserted in the membrane.

### IL-1 receptors

IL-1α and IL-1β signal through the same IL-1Rs, which belong to the immunoglobulin (Ig) supergene family and are abundantly expressed on many cell types. IL-1R of type I (IL-1R1) (80 kDa) is a signaling receptor, whereas the IL-1R of type II (IL-1R2) (68 kDa) serves as a decoy target, acting to reduce excessive amounts of IL-1 [reviewed in Ref. (1–7)]. Following the binding of IL-1 to IL-1R1, a second chain, i.e., the IL-1R acceptor protein (IL-1RAcP) is recruited. This heterodimeric complex triggers IL-1 signaling by activating the IL-1 receptor-associated kinase (IRAK) and ultimately leads to activation of NF-κB and its target genes. On the contrary, IL-1R2 and the IL-1Ra do not form this heterodimeric complex with the IL-1RAcP and therefore do not recruit IRAK. Signaling through surface IL-1R1 represents an evolutionary conserved mechanism homologous to the TLR pathway.

## Major Biological Activities of IL-1

### Effects of IL-1 on inflammatory responses

IL-1α and IL-1β are defined as “alarm cytokines” that are secreted by macrophages and initiate inflammatory responses, by inducing a cascade of other pro-inflammatory genes [reviewed in Ref. (1–7)]. Of major importance are cyclooxygenase type 2 (COX-2), inducible nitric oxide synthase (iNOS), chemokines/cytokines, and matrix metalloproteinases (MMPs). The IL-1 molecules stimulate their own and each other’s production; this represents an important amplification loop of the inflammatory response. Also, IL-1 increases the expression of integrins on endothelial cells, stromal cells, and leukocytes and thereby promotes cell infiltration into inflamed tissues.

Recently, the unique alarmin function of ProIL-1α in sterile inflammation has been described by us and others (26–30). In tissue cells, such as epithelial cells, endothelial cells, and fibroblasts, ProIL-1α is located in the cytosol and nucleus. Upon stress induction, expression of ProIL-1α increases and it translocates into the nucleus, where it is bound to chromatin in a highly dynamic manner. In stressed cells, i.e., in hypoxic conditions, ProIL-1α expression is initially increased, with the involvement of the transcription factors HIF-1α and HIF-1β. Upon necrotic cell death, ProIL-1α is released and induces inflammation (31). However, following apoptotic death, the mobility of IL-1α is greatly reduced, it concentrates in dense nuclear foci and is not released into the environment (27). This represents a novel mechanism that explains why inflammatory responses are not generated upon apoptosis. Necrotic cells lacking IL-1α failed to induce this early inflammatory response. The early infiltrate found in Matrigel plugs containing lysates of necrotic cells consists mainly of neutrophils and myeloid progenitor cells; recruitment of cells is via IL-1R1 signaling (27, 29). Macrophages infiltrate such Matrigel plugs at later times and they actively secrete IL-1β, which terminates inflammation, resulting in wound healing and restoration of tissue homeostasis (29). These results indicate significant differences in the capacity of the major IL-1 agonistic molecules to alarm inflammatory cells, thereby controlling the inflammatory response. Recently, a novel mechanism to control IL-1α activity in necrotic cells has been described by Zheng et al. (21) and reviewed in Ref. (22). Zheng found that under normal conditions, IL-1α is synthesized as a p33 precursor that is sequestered in the cytosol by IL-1R2 where it cannot be cleaved by proteases or activate IL-1R1 signaling. However, after inflammasome activation, IL-1R2 can be cleaved by caspase-1 and ProIL-1α can be released and further processed by calpain to the highly active p17 mature IL-1α form. Previously, it was thought that ProIL-1α and mature IL-1α are active to the same extent. However, this study demonstrated that the affinity of mature IL-1α to IL-1R1 is about 50 times higher than that of ProIL-1α; in accordance, their biological activity significantly differs (22). Moreover, necrosis-induced IL-1α activity is tightly controlled in a cell type-specific manner (21). Thus, in cell types with a silent necrotic phenotype, IL-1R2 remains associated with ProIL-1α. In contrast, in cells with an inflammatory, necrotic phenotype, IL-1R2 is either absent or caspase-1 is activated before necrosis. Overall, the extent of inflammation in damaged tissues depends on the concentration of cleaved IL-1α, as well as the local expression of IL-1R1. This control mechanism evolved in order to prevent exacerbation of inflammation induced by necrotic cells in tissues with limited regenerative capacity, such as kidney, heart, and brain (21, 32). These findings suggested that sterile inflammation can occur even without activation of IL-1β. Other studies have also demonstrated inflammasome-dependent IL-1α release in sterile inflammation, which may further lead to ProIL-1β expression, caspase-1-dependent processing and release [reviewed in Ref. (2, 22)].

### Effects of IL-1 on immune responses

As a pleiotropic cytokine, IL-1 has diverse potentiating effects on the proliferation, differentiation, and function of various innate (NK cells, macrophages, granulocytes etc.), as well as specific immunocompetent cells (T and B cells) [reviewed in Ref. (1–7)]. Most pronounced are the effects of IL-1 on T cell activation. Initially, IL-1 was characterized as “the classical” co-stimulatory cytokine for T cell proliferation, inducing IL-2 secretion and expression of high affinity IL-2Rs by activated T cells (33). Recent studies by the Paul group demonstrated that IL-1β induces a robust and durable expansion of naïve and memory CD4^+^ T cells (Th1, Th2, and Th17) in response to antigen stimulation and also enhances their function (34). The responding T cells must express IL-1R1 and different members of the IL-1 family were shown to activate particular STATs, which leads to the expression of relevant subset-specific transcription factors that reinforce the polarized phenotype; IL-33 and STAT5 induce Th2, IL-1β and STAT3 induces Th17 and IL-18 and STAT4 induces Th1 cells (35). Stimulatory effects of IL-1 on activation of antigen-specific CD8^+^ T cells, migration and killing were recently described (36). IL-1 can thus serve as an adjuvant in immunization, especially against weak immunogens. Indeed, in some studies, IL-1β has been characterized as an “endogenous adjuvant” that is generated following immunization with adjuvants, such as CFA and aluminum hydroxide (Alum) (37–39). The adjuvant properties of IL-1 in T cell activation possibly stem from its ability to serve as a danger signal, recruiting inflammatory cells to the site of antigen application and inducing maturation and activation of professional APCs.

### Effects of IL-1 on hematopoiesis

Multiple hemopoietic functions have been attributed to IL-1, especially to IL-1β [reviewed in Ref. (1–7)]. The *in vivo* importance of IL-1 in stimulating hematopoiesis is best demonstrated by its ability to rescue mice after lethal irradiation or chemotherapy, mainly via inducing recovery of the myeloid compartment (40). IL-1 was characterized as hemopoietin-1, a factor essential for hematopoiesis, acting by inducing the expression of receptors for colony-stimulating factors (CSFs) on primitive precursor cells (41). Of special relevance to the malignant process are the effects of IL-1 on immature Gr-1^+^CD11b^+^ myeloid cells, also termed myeloid-derived suppressor cells (MDSCs). MDSCs consist of cells committed to differentiate in the bone marrow (BM) into granulocytes, macrophages or dendritic cells [(reviewed in Ref. (42)]. In cancer or chronic inflammation, MDSCs expand in the BM in response to diverse systemic pro-inflammatory cytokines, including IL-1β; they subsequently exit the BM as immature cells and seed at sites of tumor/inflammation. They also accumulate in the spleen and lymph nodes. MDSCs remain immature and are further activated by inflammatory products to acquire immunosuppressive and pro-invasive characteristics, the latter mediated through secretion of VEGF and MMPs. MDSCs mainly consist of subpopulations of granulocyte MDSCs (G-MDSCs) and macrophage MDSCs (M-MDSCs). G-MDSCs have a limited lifespan and usually undergo apoptosis at tumor/chronic inflammation sites, while and M-MDSCs differentiate into M2 tumor-associated macrophages (TAMS). However, in some cases M-MDSCs mature into M1 anti-tumor macrophages.

## Similarities and Differences Between IL-1α and IL-1β

Mature secreted IL-1α and IL-1β as well as Pro IL-1α bind to the same receptors and exert the same biological activities, although changes in the affinity of binding of these ligands to IL-1R1 have been described. Generally, IL-1β, due to its secreted nature, has been considered to be the major IL-1 pro-inflammatory molecule and only few comparative studies on *in vivo* biological functions of both IL-1 agonistic molecules have been performed. However, some characteristics of IL-1α and IL-1β differ dramatically [reviewed in Ref. (1–7)]. IL-1β is not present in homeostatic conditions; it is induced and secreted only upon inflammatory signals and its secretion is tightly controlled at the levels of transcription, mRNA stability, translation, and processing. On the other hand, IL-1α is present in the cytosol, nucleus, or cell membrane in homeostatic states, as well as in inflammation, when its expression is upregulated. Importantly, IL-1α is only rarely secreted by living cells and in most cases is undetectable in body fluids. Previously, we demonstrated that *in vivo*, in steady-state homeostasis and in inflammation, IL-1α and IL-1β are differentially expressed in tissues, possibly pointing to their different physiological roles (32, 43).

IL-1α and IL-1β differ in the sub-cellular compartments in which they are active. IL-1β is solely active as an extracellular secreted product, while its precursor is inactive and there is no membrane-associated form of IL-1β. On the other hand, IL-1α is mainly present in its cell-associated forms (ProIL-1α, IL-1NTP and membrane-associated forms), but is only marginally secreted in its mature from, with the exception of activated myeloid cells [reviewed in Ref. (1–3)]. Very little is known about the biological activity of IL-1NTP. Intracellular forms of IL-1α were shown to translocate to the nucleus, due to a nuclear localization sequence (NLS) located within the structure of ProIL-1α and IL-1NTP, but lacking in the mature form of IL-1α. In cells that express ProIL-1α, but do not secrete it, the cytokine possibly acts in an intracrine manner from within the cell, without the need to be secreted, via signaling pathways that are not yet fully characterized. We have hypothesized that intracellular forms of IL-1α evolved as intracellular effector molecules undertaking important homeostatic regulatory functions beyond the realm of immunity and inflammation. These include effects on gene expression, cell growth, and differentiation, which were demonstrated in tissue-resident cells, such as endothelial cells, fibroblasts, smooth muscle cells, keratinocytes, epithelial cells, and brown fat cells [reviewed in Ref. (1–3)]. Thus, IL-1α belongs to a group of “dual function” cytokines (i.e., HMBG1 and IL-33) that are expressed in the cytosol and can enter the nucleus, where they perform homeostatic functions, but upon cell necrosis, they are released into the microenvironment and serve as alarmins by inducing inflammation [reviewed in Ref. (44, 45)].

We have hypothesized that the localization of the IL-1 molecules in the context of the producing cell and its microenvironment dictates their biological function in normal homeostasis and also in the malignant process [reviewed in Ref. (1, 46, 47)]. Thus, as will be shown below, membrane-associated IL-1α is immunostimulatory, while cytosolic ProIL-1α controls intracrine homeostatic functions (27, 48). However, when cytosolic ProIL-1α is released from damaged cells, it acts as an alarmin to initiate inflammation. Secreted IL-1 (mainly IL-1β), at low local doses, induces limited inflammatory responses followed by activation of specific immune mechanisms, while at high doses, broad inflammation accompanied by tissue-damage and tumor invasiveness are observed.

## Differential Activities of IL-1α and IL-1β in the Malignant Process

In the tumor arena, IL-1 is an abundant cytokine that can be secreted by malignant or microenvironment cells and affect inflammation, hematopoiesis, and immunity. It is involved in all phases of the malignant process, such as tumorigenesis, tumor invasiveness and progression, as well as activation/suppression of anti-tumor immunity. In the malignant process, the target cells of IL-1 can include pre-malignant or malignant cells, as well as cells of the microenvironment that are activated by exogenous IL-1, usually to produce inflammatory mediators that promote invasiveness. In tumorigenesis, IL-1 of microenvironment origin can propagate initial mutations by ROS or NO, produced by phagocytes, other microenvironment cells, or the mutated cells. It can then rescue initiated cells from apoptosis, enable their proliferation and further accumulation of mutations, ultimately leading to a malignant phenotype. IL-1 can then potentiate the invasiveness of malignant cells through stimulation of growth factors, angiogenesis, and tumor cell motility, leading to metastasis. In some cases, IL-1 can also enhance the immunogenicity of malignant cells and consequently reduce tumor invasiveness. As IL-1 is an upstream cytokine, its effects on the malignant process may be direct or indirect, being mediated by cytokines/mediators that it induces. Thus, at tumor sites, IL-1 induces a local cytokine network that is determined by the array of expressed cytokines, their relative concentrations, and the expression pattern of their receptors. This cytokine network dictates the dominant “net cytokine effect” and it fluctuates at various phases of tumor development. We have thoroughly studied the role of IL-1α and IL-1β in the malignant process and have shown that in many cases they perform distinct functions. The results of these studies are summarized below.

## Effects of IL-1 on Tumorigenesis

Tumorigenesis encompasses the *in vivo* induction of tumors cells by carcinogens or oncogenes, as well as *in vitro* transformation of normal or immortalized non-tumor forming cells into overt malignant cells that are capable of tumor formation in mice.

### Constitutive expression of IL-1β in the stomach can result in tumorigenesis

The overexpression of human IL-1β fused to a signal peptide (ssIL-1β), in mouse stomach epithelial cells leads to development of spontaneous gastric inflammation, pre-neoplastic lesions, and in some cases, tumors. Thus, secreted IL-1β serves as both an initiator and a tumor promoter (49). This correlates with recruitment of MDSCs to the stomach and their *in situ* activation through the IL-1R1/NF-κB pathway. Gastric pre-neoplasia and MDSC mobilization were inhibited by the IL-1Ra. In this system, overexpressed levels of IL-1β in the stomach induced a strong local inflammatory response, driven by continuous NF-κB activation, which promoted extensive hyperplasia and subsequent tumorigenesis. The ssIL-1β construct, driven by the elastase promoter in the pancreas, resulted in severe chronic pancreatitis; the severity of lesions and local inflammation correlated to the extent of IL-1β expression (50). In this system, older mice developed acinar-ductal metaplasia, but no tumor development was observed.

### Local expression of IL-1β is involved in chemical carcinogenesis

We have demonstrated the role of host-derived IL-1 molecules on susceptibility to chemical carcinogenesis induced by 3-methylcholanthrene (3-MCA), which acts both as an initiator and a tumor promoter, using a battery of IL-1 KO mice [IL-1α^−/−^, IL-1β^−/−^, IL-1α/β^−/−^(double KO mice), or IL-1Ra^−/−^mice] in comparison to wild-type (WT) mice (51). We found that deficiency of IL-1β leads to delayed 3-MCA-induced fibrosarcoma development. In mice deficient in IL-1β, tumors appeared only after a prolonged lag period and developed only in part of the treated mice. In WT and IL-1α^−/−^ mice, carcinogenesis patterns were similar and all mice developed tumors. In IL-1Ra^−/−^ mice, in which unattenuated levels of the IL-1 molecules are present, tumor development was more rapid than in WT mice. An early inflammatory response consisting of neutrophils was detected as early as 10 days after carcinogen injection. At later times, when tumor cells are already apparent, the local infiltrate consisted mainly of macrophages, which is consistent with the role of macrophages in tumor progression (52–54). Patterns of inflammation correlated with tumor development. Thus, in mice deficient in IL-1β, almost no inflammatory response was observed during tumor development, lack of IL-1α did not impair inflammation as compared to WT mice, while an heightened inflammatory response was evident in IL-1Ra KO mice. These results indicated for the first time that 3-MCA-induced carcinogenesis is inflammation-dependent, as previously it had been suggested that tumor development is controlled by immune surveillance mechanisms that eliminate the arising malignant cells [reviewed in Ref. (55)].

### Constitutive expression of IL-1α in the skin initiates benign skin papillomas

The role of wound healing in tumorigenesis has recently been reviewed (56, 57). In mice specifically over-expressing a transgene of MAPK kinase 1 in the suprabasal layer of the skin, where non-proliferating but differentiating keratinocytes reside, keratinocyte-derived IL-1α initiates wound-induced papilloma formation (58). In such mice, hyperproliferative epidermis and a chronic inflammatory infiltrate were observed. However, papillomas developed only upon skin wounding. In normal keratinocytes IL-1α is present constitutively in the cytosol and its expression increases in cells expressing the MAPK kinase 1 transgene, but it is not released into the microenvironment. Following a skin wound, IL-1α is released from dying cells and activates an inflammatory response in the suprabasal layer by infiltration of macrophages and γδT. Subsequently, immature keratinocytes are recruited into the suprabasal layer where they proliferate, leading to the development of benign tumors. Treatment with dexamethasone, which impairs cytokine production and cell infiltration, or with IL-1Ra, dramatically reduced the local inflammation and papilloma formation. IL-1β is not involved in wound-induced papilloma formation.

### The alarmin function of IL-1α contributes to carcinogen-induced liver carcinogenesis

In a model of diethylnitrosamine (DEN)-induced liver carcinogenesis in mice lacking p38α in hepatocytes, the activity of the carcinogen is enhanced as compared to that in WT mice. This is due to enhanced ROS accumulation in hepatocytes, hepatocyte cell death, and liver damage, which ultimately culminates in carcinogenesis. IL-1α is homeostatically expressed in hepatocytes and is released from dying hepatocytes; it stimulates local inflammatory responses, as well as a compensatory proliferative response that characterizes the regenerating liver. These events contribute to the development of hepatocellular carcinoma (HCC) (59). Inhibition of IL-1α or ablation of IL-1R1 prevents HCC development. In this model, IL-1α-induced IL-6 activates STAT3 and promotes liver regeneration and tumor outgrowth (60, 61). Similar effects were observed during gastric neoplasia in mice with a conditional knockout of IKKβ (lack of NF-κB signaling) in gastric epithelial cells (GECs) after exposure to stress induced by Helicobacter felis infection or ionizing irradiation (62). This resulted in a local accentuated inflammatory response, manifested by increased ROS production, tissue damage, apoptosis followed by cell necrosis, and release of IL-1α from GECs. This inflammatory response ultimately resulted in rapid progression to gastric pre-neoplasia, which was inhibited by blocking IL-1 signaling.

### Endogenous expression of IL-1α in oncogene-transformed cells facilitates their invasive potential

Several oncogenes, including Ras, Myc, and Ret not only mediate neoplastic transformation, but also activate intrinsically inflammatory cytokines that establish the pro-invasive tumor microenvironment [reviewed in Ref. (63)]. Thus, in a model of two-stage skin carcinogenesis (DMBA/TPA), mutated Ras appears early in initiated cells, whereas inflammation induces tumor promotion. Mice lacking IL-1R1 or MyD88 are less sensitive to topical skin carcinogenesis (64). The role of IL-1 in acquiring the malignant phenotype of Ras transformed primary keratinocytes was studied. It was shown that Ras-transduced keratinocytes concomitantly express IL-1α that acts in an autocrine loop together with IL-1R and MyD88. This loop controls defects in keratinocyte differentiation that are observed in papillomas and skin malignancies, as well as NF-κB activation. Treatment with IL-1Ra, reversed the differentiation defects and inhibited pro-inflammatory gene expression in keratinocytes, indicating that IL-1α is secreted from the cells and then activates them in an autocrine manner through IL-1R1 (64). Similar findings were found in a model of pancreatic ductal carcinoma (PDAC), in which constitutive K-ras and NF-κB activation are characteristic. In this model, early mutation activation of K-ras occurs, but the pathways leading to NF-κB activation are not clear. Mutated Ras induces an IL-1α-dependent mechanism that leads to constitutive NF-κB activation and tumor promotion (65). Thus, mutated K-ras induces AP-1 that subsequently activates IL-1α expression/secretion, which further leads to NF-κB activation in an autocrine manner, resulting in expression of its target genes, ultimately leading to PDAC development and invasiveness. Constitutive NF-κB activity is mediated by feed-forward loops activated by two NF-κB target genes: IL-1α and p62 – an adaptor protein that prolongs NF-κB activation by intervening in K63-polyubiquitination, which are both constitutively induced as a result of NF-κB activation and further fuel its constant activation. IL-1β is not involved in this regulatory circuit. These results substantiate the significance of the NF-κB/IKKβ pathway as a key link between inflammation and cancer, inducing pro-inflammatory cytokines in myeloid cells and anti-apoptotic pathways in epithelial cells [reviewed in Ref. (66, 67)]. The mechanisms of unique induction of either IL-1α or IL-1β in initiated cells are still unknown and are possibly dependent on the cell type.

## Tumor Cell- or Host-Derived IL-1α Preferentially Activates Anti-Tumor Cell Immunity

### Anti-tumor effects of cell-associated IL-1α

We demonstrated the anti-tumor effects of IL-1α expression by malignant cells in different experimental systems, using oncogene-transformed fibroblasts that constitutively express IL-1α, possibly due to alterations in the control of IL-1α expression induced by the oncogene. Fibrosarcoma cells were transfected with cDNA of ProIL-1α and lymphoma cells were induced to express IL-1α in a transient manner, following *in vitro* activation of the cells with immunomodulators/cytokines (68–73). In these cell lines, IL-1α is expressed in the cytosol or on the membrane, but is not secreted. In such cells, IL-1β is not expressed. IL-1α-expressing tumor cells usually fail to cause tumor development in intact mice but if tumors occur, they subsequently regress. Tumor regression in this instance is mainly mediated by CD8^+^ T cells, with some contribution of NK cells and macrophages. Regression of tumors from IL-1α-positive fibrosarcoma cells does not require activation of CD4^+^ T cells, which suggests that cell-associated IL-1α may act as a membrane-associated co-stimulatory molecule or focused adjuvant that directly activates CD8^+^ T cells. Tumor cell-associated IL-1α also potentiates antigen presentation by the malignant cells themselves, possibly through IFNγ-induced MHC class II expression, and also via cross-presentation by professional APCs. Tumor regression induces a long-term specific immune memory that protects mice against a challenge with violent parental cells.

Tumor cell-associated IL-1α was shown to be effective in tumor cell vaccines used to intervene in the growth of tumor cells of the corresponding violent line (non-IL-1 expressing). Thus, the vaccine induced regression of violent fibrosarcomas when applied at a critical “therapeutic window” 5–10 days (single application of Mitomycin-C-treated tumor cells) after inoculation of the malignant cells (70).

The “natural” membrane-associated form of IL-1α is important for exerting anti-tumor effects, as it acts as an adhesion-molecule, allowing efficient cell-to-cell interactions between malignant and immune effector cells that bear IL-1Rs, which enables better killing. Membrane IL-1α is also effective as a focused adjuvant that efficiently acts at low levels of expression, below those which are toxic to the host. Other studies have also emphasized the effectiveness of membrane-associated cytokines expressed on engineered tumor cells (i.e., IFNγ, GM-CSF, M-CSF, TNFα, and IL-12) (74–76).

### Host-derived IL-1α is essential for immunoediting during carcinogenesis

We have shown that patterns of IL-1 expression in the microenvironment can affect the immunogenicity of the arising malignant cells during tumorigenesis. Immunoediting has mainly been studied in the process of experimental carcinogenesis induced by 3-MCA. Thus, we have shown that transplantable 3-MCA-induced fibrosarcoma cell lines obtained from IL-1α^−/−^ mice failed to induce tumors in immune intact mice, whereas in sublethally irradiated mice, tumors do develop (77). This is despite the fact that tumor incidence and the nature of the local inflammatory response were comparable in 3-MCA-treated IL-1α^−/−^ mice and WT mice. Impaired immunoediting occurs in 3-MCA-induced tumors in various immunodeficient mice that lack critical components essential for the development of anti-tumor cell immunity. These include mice lacking immunosurveillance cells, such as Rag2^−/−^ mice, which lack T cells and B cells, nude mice, CD1d^−/−^ mice, which lack CD1d-restricted T cells, and Ja18^−/−^ mice, lacking semi-invariant NKT cells or mice deficient in cytokines critical for anti-tumor immunity, such as IFNγ and IL-12 [reviewed in Ref. (55, 78)]. IL-1α can now be added to the list of cytokines of importance in immunoediting. It is not yet known which form of IL-1α (secreted or cell-associated) is involved in the process of immunoediting. The process of immunoediting in immunodeficient hosts allows the survival of malignant cell variants, which are “universally immunogenic,” as they express surface adhesion or co-stimulatory molecules (i.e., ICAM-1 or 2, LFA-1 or 3, CD1d, VLA-4, B7 etc.) that are essential for the development of anti-tumor immunity. Thus, IL-1α^−/−^ cell lines were shown to express more surface MHC class I molecules, co-stimulatory molecules (i.e., B7.1 and B7.2) as well as adhesion molecules, such as L-selectin and ICAM-1, as compared to fibrosarcoma cells from WT mice. IL-1α^−/−^ immunogenic cells are rejected in intact mice by conventional innate and specific anti-tumor immune effector cells, including NK cells, as well as by CD4^+^ and CD8^+^ T cells. Immune impairments in IL-1α^−/−^ mice, including in NK development and in the killing capacity of NK cells, LAK cells, and CTLs, were characterized. The role of IL-1α in activation of immunosurveillance cells was shown in transgenic mice over-expressing IL-1α in the skin (79). In such mice, DMBA/TPA treatment induced skin tumors at very low incidence compared to WT mice, due to the rapid eradication of arising malignant cells by innate effector cells activated by local IL-1α in the skin. We hypothesize that IL-1α in the tumor microenvironment is immunostimulatory, rather that inflammatory, due to its localization on the surface of cells and its limited secretion, as compared to IL-1β. However, when IL-1β levels in tissues are limited, it can also be immunostimulatory, as will be shown below.

## Tumor Cell- or Host-Derived IL-1β is Involved in Tumor Invasiveness

### IL-1β secreted by malignant cells increases their invasive potential and induces tumor-mediated immune suppression

To assess effects of tumor cell-associated IL-1β on tumorigenicity patterns, we transfected violent fibrosarcoma cells with constructs bearing the cDNAs of the mature form of IL-1β or the mature form of IL-1β ligated to a signal sequence (ssIL-1β), to induce potent secretion of IL-1β through the endoplasmic reticulum-Golgi pathway (80, 81). We found that IL-1β- and ssIL-1β-transfected fibrosarcoma tumors were more invasive than the violent parental cells or mock-transfected cells. The invasiveness of the malignant cells correlated with the amount of IL-1β that was secreted by them. In addition, only the ssIL-1β transfectants, which secrete relatively high levels of the cytokine, exhibited a metastatic potential. Enhanced angiogenesis patterns, as evidenced by high vessel density in tumors and increased secretion of VEGF by the malignant cells, were observed in tumors secreting IL-1β. Similar observations were described in other experimental systems using IL-1β-transfected tumor cells (82–84). No anti-tumor effector cells or cytokines that potentiate anti-tumor immunity (i.e., IFNγ and IL-2) could be detected in spleens of mice injected with fibrosarcoma cells transfected with IL-1β or ssIL-1β or with the violent parental cells. In contrast, effective anti-tumor cell immune responses were observed in mice injected with fibrosarcoma cells transfected with ProIL-1α, as indicated above.

Further studies have shown that general anergy develops in mice bearing tumors of IL-1β secreting cells, mediated by MDSCs (80, 82, 85). Resection of large tumors of IL-1β secreting cells completely restored immune reactivity and reversed the MDSC response within 7–10 days. Treatment of tumor-bearing mice with the IL-1Ra reduced tumor growth and attenuated the MDSC response.

In spite of tumor-mediated suppression, resection of large tumors of IL-1β secreting cells, followed by a challenge (2 months after tumor resection) with the violent parental cells induced resistance in mice; protection was not observed in mice bearing tumors of mock-transfected fibrosarcoma cells. Thus, in mice bearing tumors of IL-1β secreting cells, anti-tumor cell specific immunity is activated, due to the adjuvant-like effects of IL-1β; however, protective immunity is not manifested, due to suppression of immune effector mechanisms. It is notable that when tumor cells expressing membrane-associated IL-1α are injected into mice, anti-tumor immune responses occur without concomitant tumor-mediated suppression and thus the malignant cells are rejected.

## Tumor-Mediated Angiogenesis is Largely Stimulated by Microenvironment IL-1β

We have studied in detail the role of IL-1β in tumor-mediated angiogenesis, which is almost non-existent upon injection of tumor cells into IL-1β KO mice or following neutralization of IL-1β in WT mice (86, 87). Inflammation usually accompanies tumor-mediated angiogenesis. We have used B16 melanoma cells encapsulated in Matrigel plugs, in order to characterize cell/cytokine interactions in the early angiogenic response (88). We have characterized a newly described auto-induction circuit in which IL-1β and VEGF interact and induce each other. Tumor-mediated angiogenesis is inhibited if either IL-1β or VEGF are neutralized and it does not occur in IL-1β KO mice. The IL-1β and VEGF circuit acts via interactions between BM-derived VEGFR1^+^/IL-1R1^+^ immature myeloid cells (MDSCs) and tissue-resident endothelial cells. Myeloid cells do not directly stimulate endothelial cells for migration and subsequent blood vessel formation. However, myeloid cells produce IL-1β and a network of pro-inflammatory cytokines/molecules, which subsequently activate resting endothelial cells to produce VEGF, as well as other direct pro-angiogenic factors. Subsequently, VEGF activates endothelial cells for blood vessel formation. IL-1β thus provides the inflammatory microenvironment for angiogenesis and tumor progression. We have shown that IL-1β inhibition stably reduces tumor growth, by limiting inflammation and by inducing the maturation of MDSCs into M1 macrophages, which do not promote tumor invasiveness and can be cytotoxic/cytostatic for tumor cells and can also serve as APCs that induce anti-tumor immunity. Thus, this study has characterized IL-1β as a major mediator in the tumor microenvironment that recruits MDSCs and also controls their immature pro-invasive and immunosuppressive state; ablation of IL-1β alters the pro-tumor microenvironment into an anti-tumor one.

### Microenvironment IL-1 activates cancer stem cells

In murine tumor models and in cancer patients, it was shown that IL-1β increases tumor invasiveness [reviewed in Ref. (1, 46, 47)]. Recently, direct effects of IL-1β on cancer stem cells (CSCs) or the niche that favors CSC formation were described. In *in vitro* studies, recombinant IL-1β increased the sphere forming capacity of CSCs and enhanced expression of stemness genes (i.e., Bmi1 and Nestin), as well as Zeb1 that is an important regulator of EMT and self-renewal (89). Furthermore, the Weinberg group has described a circuit in which carcinoma-derived IL-1 creates the niche for the transition of “regular tumor cells” into CSCs (90). Thus, in the tumor microenvironment, carcinoma-derived IL-1 (IL-1α and IL-1β) activates mesenchymal stem cells (MSCs) to produce PGE_2_ and other cytokines, such as IL-6, IL-8, Gro-α, and RANTES that in turn act on the carcinoma cells and induce activation of β-catenin and transition into CSCs. These effects were largely neutralized by the IL-1Ra or siRNAs against IL-1α and IL-1β. Thus, IL-1 in the tumor microenvironment can support development and expansion of CSCs and thus amplify the malignant process and support metastasis formation.

### Anti-tumor effects of microenvironment IL-1β in mice with minimal residual disease

Elegant studies by the Zitvogel group demonstrated that tissue-damage following cancer treatment with some chemotherapeutical drugs activates DCs in the tumor microenvironment to present tumor antigens and further stimulate anti-tumor immunity that synergizes with the chemotherapy (91). In the milieu of anthracycline-treated tumors, the NLRP3 inflammasome is activated and stimulates IL-1β production that is essential for activating IFNγ producing CD8^+^ T cells. In addition, patients with breast cancer with a loss-of-function allele of P2X7R, which is essential for activation of the NLRP3 inflammasome and IL-1β processing/secretion, develop a more rapid metastatic disease than individuals with the normal allele. This may represent a unique scenario in which a low tumor burden, possibly accompanied by relative low levels of IL-1β in the microenvironment could activate local immunity. These results will hopefully open new avenues for use of IL-1β, and possibly also IL-1α, in cancer immunotherapy in tumor-debulked patients.

## Interactions between Tumor Cell- and Microenvironment-Derived IL-1 in the Control of Tumor Invasiveness of 3-MCA-Induced Tumor Cell Lines

In the tumor microenvironment, interactions between IL-1 derived from the malignant cells or from inflammatory cells interact and determine the invasive potential of the tumor. Transplantation assays, in which 3-MCA-induced fibrosarcoma cell lines that were derived from WT or IL-1 KO mice were injected into the same strains of mice, enabled us to define the role of IL-1 expressed by the malignant cells or the microenvironment in tumor progression (51, 77, 92–94).

3-MCA-induced fibrosarcoma cell lines from WT mice manifested low invasiveness in IL-1 deficient mice, intermediate invasiveness in WT mice, and high invasiveness in IL-1Ra^−/−^ mice, pointing to the importance of microenvironment-derived IL-1 in tumor progression. The contribution of tumor cell-derived IL-1 was demonstrated following injection of 3-MCA fibrosarcoma cell lines from IL-1 KO mice into WT mice. Thus, fibrosarcoma cells obtained from mice deficient in IL-1β failed to grow in WT mice, due to their inability to recruit a local inflammatory response that is essential for tumor invasiveness. Furthermore, invasive 3-MCA-induced fibrosarcoma cells from IL-1Ra KO mice were only weakly tumorigenic in IL-1 deficient mice (92–94). We suggest that initially, upon injection of tumor cells into mice, the malignant cells express relatively small amounts of IL-1 that subsequently induces broad inflammation mediated by infiltrating cells, ultimately leading to tumor invasiveness. In malignant cells, IL-1 can be constitutively expressed due to oncogene activation or it can be induced by danger signals in the microenvironment. IL-1 of the microenvironment is critical to induce tumor outgrowth and progression leading to invasiveness of 3-MCA fibrosarcomas.

In naturally occurring tumor cells, both IL-1 molecules can be expressed and interact. The expression of IL-1α in 3-MCA-induced tumor cells from IL-1β KO mice concomitantly activated a strong T helper and CTL response, which also contributed to the reduced *in vivo* growth of these cells in WT mice. When cell lines from IL-1α KO mice were injected into mice, progressive tumor growth occurred, due to the pro-invasive and immunosuppressive effects of tumor cell-derived IL-1β and the absence of immunostimulatory effects of tumor cell-associated IL-1α that is missing in these cells. Cell lines that originated in 3-MCA-treated IL-1Ra^−/−^ mice were very invasive and metastatic, compared to cell lines originating in WT mice, due to high-unattenuated levels of IL-1 expression in the malignant cells, which facilitates their invasiveness and also promotes immunosuppressive mechanisms. Thus, fibrosarcoma cell-derived IL-1α and IL-1β do not act in concert and each IL-1 molecule has unique effects on tumor invasiveness or on anti-tumor cell immune responses. At tumor sites, immunosuppressive effector cells, induced by excessive expression of IL-1β inhibit or mask the induction/function of anti-tumor immunosurveillance induced by tumor cell-derived IL-1α. At tumor sites, effects of tumor-derived IL-1 and host-derived IL-1 interact and modulate tumor progression. Elucidation of these interaction patterns should enable better understanding of the overall role of IL-1 in the malignant process.

## Complex Effects of IL-1 on the Invasiveness of Human Tumors

In experimental tumor models in WT mice and in cancer patients, increased local levels of IL-1 at tumor sites usually correlate with tumor invasiveness and a bad prognosis [reviewed in Ref. (1, 46, 47)]. Very little is known about interactions between IL-1α and IL-1β expressed at tumor sites by either the malignant or microenvironment cells. Most studies usually assess only one of the IL-1 agonistic molecules and do not discriminate between patterns of its expression in the malignant cells versus the microenvironment. The levels of IL-1 expression at tumor sites are also not compared to homeostatic levels in the given organ. Thus, insufficient characterization of IL-1 at tumor sites has led to some inconsistencies concerning the impact of the concerted action of IL-1α and IL-1β on the malignant process. For example, Okamoto et al. showed constitutively active NLRP3 inflammasome and IL-1β secretion in melanoma cell lines derived from late stage patients, where selection for cells expressing invasiveness-promoting molecules had already occurred. This can also explain the increased invasive phenotype of progressive melanoma tumors (95). In a different study on melanoma patients performed by the Grimm group, IL-1α was expressed in most primary tumors (98%) and approximately half (55%) of metastases. IL-1α was also expressed in 73% of inspected nevi. IL-1β was expressed in approximately 10% of primary or metastatic melanoma samples, but its expression strongly correlated with IL-1α expression (96). The mechanisms of preferential IL-1α expression in human melanomas are not known. By using cell lines obtained from human melanoma patients, typical molecular pathways of inflammation, including secretion of ROS, NO, COX-2, as well as NF-κB and c-Jun activation, were observed in malignant melanoma cells upon activation by endogenous IL-1, which also promotes *in vitro* cell proliferation. These effects were most pronounced in cell lines producing significant amounts of IL-1 and were abrogated by antibodies against IL-1R1 or siRNA of IL-1α and IL-1β. Furthermore, blocking IL-1 signaling in melanoma cell lines induced autophagy, which might further lead to cell death. IL-1 secreted by melanoma cells also affects recruitment and activation of inflammatory cells at tumor sites, which contribute to invasiveness. The correlation between IL-1 expression in tumors and its secretion is still unknown and awaits further investigation.

## Future Prospects for IL-1 Manipulation in Anti-Tumor Therapies

**Figure 1:**
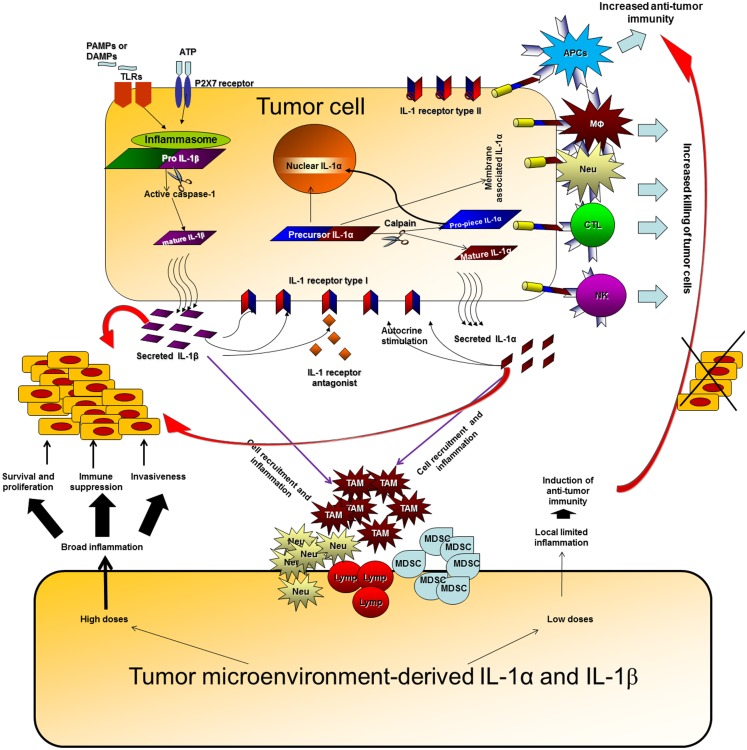
**Effects of IL-1 at tumor sites**. IL-1 can be produced at tumor sites by the malignant cells or by diverse cells in the tumor microenvironment. IL-1 generated by tumor cells can affect the malignant cells in an autocrine or paracrine manner, enabling proliferation, and invasiveness. In parallel, IL-1 secreted by malignant cells activates microenvironment residing or infiltrating cells to produce additional IL-1, which then induces a cytokine network, which further activates tumor invasiveness. High doses of IL-1 at tumor sites usually result in an invasive potential and immunosuppression. Expression of IL-1α on the membrane of malignant cells increases their immunogenicity and leads to induction of efficient anti-tumor responses. Membrane IL-1α expressed on infiltrating cells possibly also promotes the development of anti-tumor cell immunity. Low levels of IL-1 at tumor sites at early stages of tumor development or upon IL-1 attenuation, usually result in efficient anti-tumor immunity, in the absence of immunosuppression mainly mediated by IL-1-induced MDSCs and also Tregs. When immunosuppression is evident at tumor sites, it hinders the development or masks the function of anti-tumor immunity and thus invasive growth results. Host- and tumor cell-derived IL-1 induce each other and together fuel the local, and sometimes systemic, inflammatory response. Intracellular ProIL-1α in tumor cells induces intracrine functions following translocation into the nucleus. These are related to survival, proliferation, or gene expression; however, they were not sufficiently characterized in the context of the malignant process.

The network of cytokines and immune/inflammatory cells in the tumor microenvironment controls the fate of the malignant process. In the tumor microenvironment, the balance between the “wound healing” type of inflammation, which promotes tumor progression and immune escape, and “favorable” limited inflammatory responses, in which professional APCs are activated and induce anti-tumor adaptive immunity, determines the direction of the malignant process [reviewed in Ref. (97–101)]. Due to the plethora of activities of IL-1 in the malignant process and its dominant role in determining local cytokine networks at tumor sites, neutralization of IL-1 as a single target molecule has potential to tilt the balance between destructive inflammation and protective anti-tumor immunity in the tumor microenvironment (Figure 1). For example, we have shown that 4T1 cells produce invasive and metastatic breast tumors which grow progressively in WT mice, whereas, they cause tumors that grow initially but later regress in IL-1 KO mice, due to the efficient induction of a CTL-mediated anti-tumor response in these mice.

Use of genetically engineered cells or mice with distinct patterns of IL-1 expression, have shown that IL-1α and IL-1β have distinct effects at tumor sites. Thus, IL-1β promotes invasiveness and immunosuppression, while IL-1α is mainly immunostimulatory. This is true for both tumor cell-derived or microenvironment-derived IL-1. Immunosuppression induced by IL-1β usually acts in a dominant manner and masks the immunostimulatory anti-tumor effects of IL-1α.

However, in “real-life” both IL-1 molecules are usually expressed and they induce each other. Further studies should establish the “IL-1 map” in individual tumors, taking into account patterns of expression, secretion, and levels of IL-1α and IL-1β in malignant or infiltrating cells. Having these data should facilitate the design of treatment protocols based on IL-1 manipulation.

IL-1 neutralization agents are available at the present time (102– 104). Thus, the IL-1Ra, also called Anakinra (Kineret; Amgen/Biovitrum) is FDA-approved and has been shown to be safe and efficient in alleviating symptoms of rheumatoid arthritis and auto-immune diseases. Characterization of the inflammasome pathway of IL-1β processing and secretion encouraged the development of novel anti-IL-1 agents that are now being tested in different clinical trials in diverse diseases with inflammatory manifestations. Some of these trials have produced initial promising results. These agents now await testing in cancer patients, once protocols are established for their integration into first-line anti-tumor therapies. Optimally, IL-1 neutralization should be most effective in patients with minimal residual disease (MRD), to prevent tumor recurrence and metastasis. In such patients, tumor-mediated immunosuppression and inflammation should be reduced and enable induction of protective anti-tumor immune responses in a microenvironment that does not favor invasiveness. These treatments may be given for extended periods, to convert MRD to a chronic state, provided that resistance to anti-IL-1 therapy does not develop. Neutralization of tumor-associated IL-1, especially IL-1β, should not be complete, in order not to compromise the immune system of patients. In addition, due to the adjuvanticity of cell-associated IL-1α, tumor cell vaccines based on constitutive or transient IL-1α expression has the potential to induce anti-tumor cell immunity in patients with MRD. In such patients, one can envision initial systemic neutralization of IL-1β followed by application of IL-1α expressing tumor cell vaccines. In conclusion, better understanding the integrative role that IL-1α and IL-1β play in animal experimental models and in cancer patients, together with “IL-1 mapping” at tumor sites, as indicated above, should pave the way for safe and efficient application of anti-IL-1 therapies at the bedside for cancer patients.

## Conflict of Interest Statement

The authors declare that the research was conducted in the absence of any commercial or financial relationships that could be construed as a potential conflict of interest.
